# An Evaluative Study of a Nurse‐Led Surgical Information Initiative for Gender Diverse Individuals Seeking Genital Surgery

**DOI:** 10.1111/jan.16532

**Published:** 2024-10-19

**Authors:** Seán Kearns, Pauline Forrester, Donal O'Shea, Karl Neff

**Affiliations:** ^1^ School of Medicine University College Dublin Dublin Ireland; ^2^ National Gender Service St Columcille's Hospital Dublin Ireland

**Keywords:** gender‐affirming surgery, nurse‐led initiative, patient education, surgical information, transgender healthcare

## Abstract

**Aim:**

To evaluate the impact of nurse‐led one‐on‐one psychoeducation sessions on gender diverse individuals seeking gender‐affirming genital surgery.

**Design:**

A quasi‐experimental, pre‐ and post‐test research design was employed to examine the impact of a nurse‐led initiative on improving patients self‐perceived knowledge and confidence pertaining to gender affirming genital surgery. The study followed the SQUIRE 2.0 (Standards for Quality Improvement Reporting Excellence) guidelines and the COREQ (Consolidated Criteria for Reporting Qualitative Research) guidelines.

**Methods:**

The curriculum for the initiative was crafted through literature reviews, expert panel engagements, multidisciplinary team input and was delivered by two specialist gender nurses.

**Results:**

The results indicated a statistically significant increase in all participants' self‐perceived knowledge and confidence scores. Furthermore, the study increased confidence in the ability to ask questions and plan for the logistical and financial aspects of surgery.

**Conclusion:**

Participants reported that the sessions were very useful, and for most, the information did not change their desire to seek surgery but did help them make more informed choices about the procedure, timing and preferred surgeon.

**Implications for Patient Care:**

The study underscores the imperative role of support networks and recommends interventions to facilitate open communication within families. The study emphasises the importance of customising healthcare approaches to align with the preferences of patients.

**Impact:**

The study addressed the need for psychoeducation sessions for individuals considering gender‐affirming genital surgery. The main findings revealed a significant increase in participants' self‐perceived knowledge and confidence, following a nurse‐led intervention. The research's impact extends to gender‐diverse individuals seeking surgery globally.

**Patient or Public Contribution:**

Four individuals who had undergone gender‐affirming surgeries contributed their perspectives to the study design, ensuring that the educational content addressed specific information needs and concerns.

**Summary**

- What does this paper contribute to the global clinical community?
  ○ This paper's findings on the effectiveness of nurse‐led psychoeducation sessions have broad applicability globally, providing a practical model for healthcare professionals (particularly nurses) in diverse settings to enhance the support and education provided to gender‐diverse individuals seeking genital surgery.

## 1 Introduction

Gender Diverse serves as an umbrella term to describe a continuously evolving spectrum of identities people may adopt when their gender identity, expression, or perception does not align with societal norms and expectations (Thorne et al. 2019). Within this expansive spectrum, terms like transgender, non‐binary and gender non‐conforming are used. For the purposes of this paper, we will use the term gender‐diverse when describing the study population.

Gender‐affirming genital surgeries can play an important role in the journey of gender‐diverse individuals, contributing significantly to their physical and emotional well‐being (Almazan and Keuroghlian 2021). These surgeries are sometimes known as ‘gender confirmation surgery’ or ‘bottom surgery’ (Berli et al. 2017). For transfeminine people, these surgeries normally involve procedures such as orchidectomy, vaginoplasty (penile inversion ± graft or intestinal conduit), clitoroplasty and labiaplasty (Coon et al. 2023; Li, Crane, and Santucci 2021; Pariser and Kim 2019).

For transmasculine people, these surgeries involve some or all of the following procedures: Hysterectomy and oophorectomy, colpectomy/colpocleisis, metoidioplasty, scrotoplasty (testicular prosthesis) and staged phalloplasty (including a combination of phallus formation, glansplasty, urethroplasty and erectile prosthesis) (Djordjevic, Stojanovic, and Bizic 2019; Lane et al. 2020; Morrison et al. 2016). These surgeries are complex and often involve multiple surgical specialties, including urology, gynaecology and plastic surgery (Berli et al. 2017).

These procedures are typically classified as elective. Not all gender‐diverse people will want or require these surgeries (Nolan, Kuhner, and Dy 2019). Some individuals do not seek surgery as they are satisfied with their genitals as they are. Others are not interested in surgery as they consider the surgical outcomes to be unsatisfactory (Rashid and Tamimy 2013). Reliable information is an important factor in making informed decisions around genital surgery. It has been established that gender diverse people often face difficulty accessing reliable information and support (van de Grift, Mullender, and Bouman 2018).

The use of preoperative educational sessions is well documented in various healthcare domains, and evidence suggests that they can reduce anxiety, fear and stress around surgical procedures (Burgess, Arundel, and Wainwright 2019; Groller 2017). They are also successful in increasing patient knowledge of the procedure (Ronco et al. 2012). However, there are few studies on the impact of preoperative educational sessions in the area of gender‐affirming genital surgeries.

Poceta et al. (2019) designed a one‐off group class‐based initiative for patients seeking vaginoplasty and phalloplasty and reported increases in knowledge about surgical options, post operative complications and recovery. While this group approach was successful, we decided to approach our education initiative with a one‐on‐one approach, with loved ones welcome to join.

We feel that in the context of supporting gender diverse patients considering genital surgery, one‐on‐one interventions led by nursing professionals offer distinct advantages. These sessions provide tailored care, addressing individual patient needs and concerns, ensuring a personalised and relevant educational experience. Additionally, the privacy and confidentiality of one‐on‐one settings create a safe space for patients to discuss sensitive topics openly, while nurses can offer vital psychological support and emotional reassurance (Sim and Waterfield 2019). This may be particularly important in the discussion of the psychosexual implications of genital surgery (Kwame and Petrucka 2021).

Nursing professionals are well‐suited to lead these interventions due to their clinical expertise, which allows them to convey complex medical information clearly. They also bring an established foundation of trust and rapport with patients, facilitating open and honest communication essential for addressing the unique challenges associated with gender‐affirming care. Nurses' involvement ensures continuity of care throughout the patient journey, and their cultural competence enhances their ability to provide effective support tailored to the patient.

The primary aim of this study is to assess the impact of nurse‐led one‐on‐one psychoeducation sessions on the process of gender diverse individuals seeking gender‐affirming genital surgery. In line with this overarching aim, the specific objectives of our research are as follows:

1. Evaluate the effectiveness of the psychoeducation information session in enhancing participants' knowledge and confidence regarding gender‐affirming genital surgery.
2. Examine the influence of the psychoeducation session on the perceived level of support needed for surgery and their overall comfort with their bodies.
3. Identify views on the usefulness of sessions and areas for potential improvement in the psychoeducation information session based on participant feedback.
4. Investigate the factors that inform the choice of surgeon among transgender and gender diverse individuals seeking gender‐affirming surgery.

### 1.1 Educational Content Design

The educational content for the nurse‐led psychoeducation sessions was crafted through a collaborative effort within the research team. To ensure its relevance and comprehensiveness, the team conducted a literature review on gender‐affirming surgeries, synthesising and simplifying the latest advancements and insights. Subsequently, the endocrinologists (K.N., D.O.S.) and nurses (S.K., P.F.) on the research team actively engaged in research team meetings to identify key areas of information essential for patients. Their clinical expertise and experience in caring for gender diverse individuals were instrumental in shaping the content.

**FIGURE 1:**
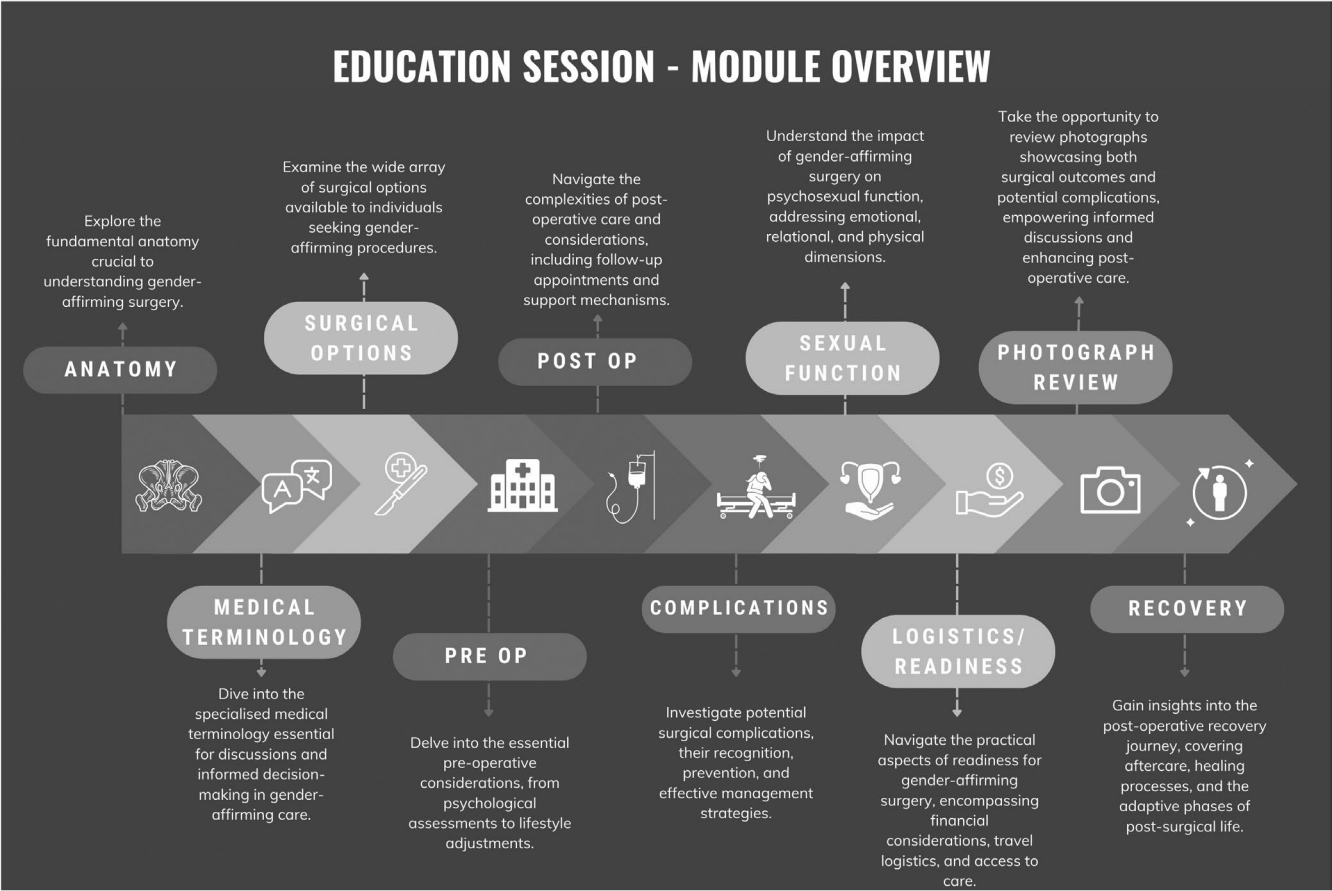
Educational session module overview. This figure visually presents the structured curriculum comprising ten modules developed collaboratively by the research team, encompassing key topics essential for gender‐diverse individuals considering gender‐affirming genital surgery.

In addition to the core team members, valuable input was sought from other members of the multidisciplinary team (MDT) involved in gender‐affirming care at our centre. After consultation, the team curated a curriculum encompassing ten topics or modules (see Figure 1), including: relevant anatomy, medical terminology, surgical options, pre‐ and post‐operative considerations, potential complications, the impact on psychosexual function, photographic outcomes, logistics/practicality related to readiness for surgery and the recovery process. This collaborative approach ensured that the educational content was well‐rounded and tailored to meet the specific needs of the patients. In addition, the team consulted with two transmasculine and two transfeminine individuals who had completed surgery to discuss their experiences. They were asked to review the surgical information content plan and provide recommendations for changes before the final sign‐off.

The content was delivered as a presentation/lecture to the individual and their loved ones. This method allowed for adaptation and questions. Participants received written copies of the slides and additional written resources during the session. The sessions took between 2 and 4 h per patient over 1–2 meetings.

## 2 Methods

### 2.1 Study Design

This study employed a quasi‐experimental, pre‐ and post‐test research design to investigate a quality improvement initiative. The initiative evaluated the impact of nurse‐led one‐on‐one psychoeducation sessions on gender diverse individuals' knowledge, confidence and decision‐making processes regarding gender‐affirming genital surgery. These factors were chosen as important by the research team and patient and public involvement group. The study followed the SQUIRE 2.0 (Standards for Quality Improvement Reporting Excellence) guidelines (Ogrinc et al. 2015).

As part of the standard care pathway, nurses within the team (S.K., P.F.) designed and provided surgical informational sessions to all patients seeking surgical information or surgery before referring individuals to the assessment team as a novel quality improvement initiative. As the entire patient cohort received the intervention, this study was classified as an observational quality improvement study, which did not meet eligibility criteria for trial registration. This initiative has been integrated into the clinical pathway with the hope of serving as resource protection, as not all recipients of the education sessions may want to pursue surgical referral and assessment after receiving more information. Furthermore, this approach equips participants with comprehensive information, enabling them to make more informed decisions regarding their assessments and the timing of their gender‐affirming journey. This study aims to evaluate this initiative and is therefore classified as a quality improvement study.

This design involved assessing participants' self‐perceived baseline knowledge and confidence levels before the intervention and comparing them with post‐intervention measurements through a survey. The study aimed to explore changes in these variables as well as participants' support for surgery, body comfort and readiness for the surgical process.

Additionally, qualitative written data were collected to investigate the factors influencing participants' choice of surgeon in the transgender and gender diverse community. The quasi‐experimental nature of this study allowed for the evaluation of the intervention's effects while considering ethical and practical constraints.

### 2.2 Patient and Public Involvement

In a commitment to patient‐centred care, the research team consulted with four patients who had previously undergone vaginoplasty (*n* = 2) and phalloplasty (*n* = 2) procedures. These individuals were invited to review the module plans and provide their first‐hand insights and feedback. Their unique perspectives as surgery recipients proved invaluable, allowing them to pinpoint specific information needs and areas of concern.

The feedback provided by these patients was carefully considered and thoughtfully embedded into the design of the educational content, ensuring that it not only met clinical standards but also resonated with the lived experiences and expectations of those seeking gender‐affirming surgeries. In particular, emphasis on the rate and impact of complications on quality of life was highlighted by participants.

### 2.3 Sampling

In this study, a combination of convenience and purposive sampling methods was employed to select participants. The rationale behind this approach was to ensure the inclusion of individuals who were actively seeking gender‐affirming genital surgery and who were already engaged with the multidisciplinary gender clinic, making them both accessible and highly relevant to the research objectives (Shorten and Moorley 2014).

### 2.4 Setting and Participants

#### 2.4.1 Context

This study was conducted within the framework of an Irish gender service for adults. The service is multidisciplinary in nature and conducts a range of psychosocial assessments related to readiness for hormone therapy and surgical interventions. In addition, the service offers endocrine and mental health services. Importantly, gender‐affirming genital surgeries are predominantly conducted overseas, given the absence of specialised surgical expertise within Ireland. The multidisciplinary team (MDT) within this service is involved in assessing suitability and readiness for these procedures, subsequently referring patients to overseas surgical centres when deemed appropriate.

#### 2.4.2 Participants

Participants were adults who attended the gender service and were over eighteen years old. They had been receiving hormone therapy through our service for at least one year and had expressed an interest in pursuing gender‐affirming genital surgery.

#### 2.4.3 Optional Research Component

While the psychoeducation sessions were a mandatory component of the clinical pathway, the research component of the study was entirely optional. Patients attending the educational sessions were informed of the research study and given the choice to participate. Emphasis was placed on ensuring voluntary participation, with individuals having the freedom to decline involvement without any impact on their clinical care.

#### 2.4.4 Consent

Participants who chose to partake in the research study were presented with detailed information about the study's objectives, procedures, potential risks and benefits in the form of a patient information leaflet (PIL). This was followed by a comprehensive discussion to address any questions or concerns. Subsequently, participants provided written informed consent, reaffirming their willingness to contribute to the study. Participants were provided ample time, specifically 20–30 min, to decide whether they wished to participate in the study. They were informed that they could withdraw at any point during the process. However, no participants chose to withdraw. The study was conducted in a hospital setting. They were invited to have a support person (friend, family member, partner etc.) attend with them.

#### 2.4.5 Data Collection

Data were collected between June 2023 and December 2023. S.K. and P.F. conducted the education sessions, distributed the patient information leaflet and undertook the informed consent process. Quantitative and qualitative data were collected through a comprehensive survey designed to capture both numerical and narrative responses about participants' experiences (Creswell and Plano Clark 2017). The survey included structured quantitative questions alongside open‐ended prompts to gather detailed feedback.

#### 2.4.6 Research Team and Reflexivity

S.K. and P.F. collected the data for this study. S.K., a male Advanced Nurse Practitioner with a PhD in gender care, and P.F., a female Surgical Clinical Nurse Specialist with an MSc, both have extensive expertise in their respective fields. Neither S.K. nor P.F. had any prior relationship with the clients involved in the study. As part of the informed consent process, patients were fully informed about the study. S.K. and P.F.'s interest in this research topic stems from their professional roles within a gender service.

#### 2.4.7 Ethics

The study received ethical approval from the St. Vincent's University Hospital Ethics Committee (RS23‐027) and the University College Dublin Ethics Committee (LS‐LR‐23‐125‐Kearns‐Neff).

#### 2.4.8 Quantitative Data Analysis

Quantitative data were analysed using descriptive and inferential statistics in SPSS. Paired t‐tests were employed to assess changes in participants' knowledge and confidence levels before and after the psychoeducation sessions. This statistical approach provided a robust measure of the intervention's self‐reported effectiveness (Field 2013).

#### 2.4.9 Qualitative Data Analysis

Qualitative data were collected through open‐ended survey questions aimed at eliciting participant responses about their experiences with the psychoeducation sessions. Participants were specifically asked to describe their understanding and feelings about the sessions and factors influencing their choice of surgeon. The qualitative data were analysed using thematic analysis, following Braun and Clarke's (2006) guidelines. This process involved coding (by S.K. & K.N.) the responses, identifying recurring themes and refining these themes to capture key insights utilising NVivo software (Braun and Clarke 2006).

#### 2.4.10 Integration of Quantitative and Qualitative Findings

The integration of quantitative and qualitative data provided a broader understanding of the intervention's impact. While the quantitative data offered measurable evidence of changes in knowledge and confidence levels, the qualitative data provided personal insights into the participants' experiences (Fetters, Curry, and Creswell 2013). It is important to note that this study is primarily quantitative‐led, with the write‐in qualitative responses providing some elements of qualitative investigation.

## 3 Results

### 3.1 Sample Characteristics

Sample characteristics: Twenty participants participated in the study (*n* = 20), and their demographics are detailed in Table 1. The sample comprised ten Assigned Male at Birth (AMAB) and ten Assigned Female at Birth (AFAB) participants. The majority (90%) of participants identified within the traditional binary gender framework (as either male or female), while 10% identified as non‐binary or otherwise gender‐diverse. In terms of age, the largest portion of the sample (65%) was between 25 and 34 years old, with 10% in the 18–24 age range, 15% between 35 and 44 years old and 5% each in the 45–54 and 65+ age ranges.

**TABLE 1:** Participant demographics.

Individual‐led variables/demographics	*N*	Percent (%)
*Total participants*	20	100
*Gender*		
AFAB‐ Trans Masculine, Trans man or man	9	45
AFAB‐ Non‐binary or gender‐diverse	1	5
AMAB‐ Trans Feminine, Trans woman or woman	9	45
AMAB‐ Non‐binary or gender‐diverse	1	5
*Age*		
18–24	2	10
25–34	13	65
35–44	3	15
45–54	1	5
65+	1	5
*Sexuality*		
Heterosexual/Straight	4	20
Gay or Lesbian	8	40
Bi‐sexual	3	15
Pansexual	2	10
Asexual	1	5
Queer	2	10
Prefer not to say	0	0
*Relationship status*		
Single, not dating	6	30
Single, dating	3	15
In a serious relationship	9	45
In serious relationships (polyamorous)	1	5
Married/domestic partnership	0	0
Prefer not to say	1	5
*Ethnicity*		
White/Caucasian	20	100
*Highest education level obtained*		
Secondary/high school diploma	3	15
College/further education course	6	30
Bachelor's degree	8	40
Master's degree	3	15
*Current household income*		
Less than €25,000	5	25
€25,000–€49,999	11	55
€50,000–€74,999	2	10
€75,000–€99,999	1	5
Unsure	1	5
Prefer not to say	—	—
*Current occupation*		
Employed (full‐time)	10	50
Student	4	20
Unemployed	2	10
Student and on disability	1	5
On disability	1	5
Retired	2	10
*Previous surgeries*		
None	10	55
Mastectomy	9	45
Hysterectomy	2	10
Facial feminisation	1	5
*Have private health insurance*		
No	14	70
Yes	5	25
Prefer not to say	1	5
*Plan to use private health insurance for GCS*		
No	10	50
Yes	—	—
Unsure	10	50

*Note:* This table provides a breakdown of the demographics of the study participants, including gender, age, sexuality, relationship status, ethnicity, education level, household income, occupation, previous surgeries and health insurance details.

All participants in the sample were Caucasian and possessed moderate levels of education. Occupational engagement levels were notably high, with 75% of the sample engaged in either education or employment. The remaining 25% were distributed among those on disability benefit (*n* = 1), retired (*n* = 2) or unemployed (*n* = 2).

Trans masculine participants were more likely to have undergone previous gender‐affirming surgeries (*n* = 11), with mastectomy being the most common surgery (*n* = 9) followed by hysterectomy (*n* = 2). One trans feminine participant had undergone facial feminisation surgery. A significant portion of participants (70%) did not possess private health insurance. Half of the participants felt they would not use insurance, while the other half expressed uncertainty regarding its utilisation.

### 3.2 Impact on Knowledge

**FIGURE 2:**
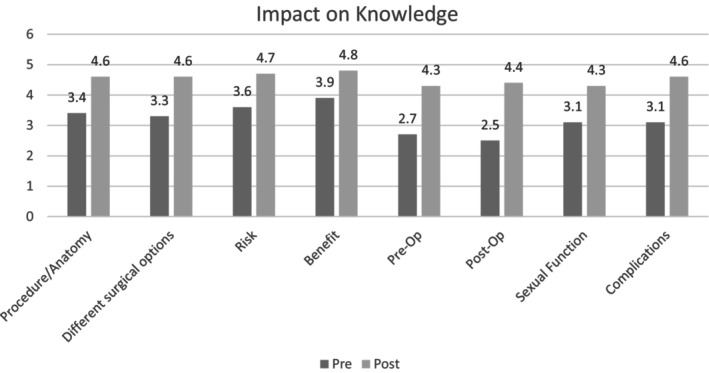
Impact on knowledge: self‐perceived understanding across surgical topics. This figure illustrates the statistically significant improvement in participants' self‐perceived knowledge scores across diverse aspects of gender‐affirming genital surgery, using a five‐point Likert scale.

There was a statistically significant increase in self‐perceived knowledge on all points assessed post‐intervention (Figure 2). These points were evaluated using a five‐point Likert scale pre‐ and post‐intervention that measured participants' understanding by asking, ‘How well do you understand… X or Y or Z’. This scale ranged from ‘Not at all’ to ‘Extremely’. The points assessed knowledge regarding: the general surgical procedure (i.e., how it is performed and what body parts are used), the different surgical choices involved, the risks of surgery, the potential benefits of surgery, the preoperative considerations (i.e., what do you need to do before surgery to prepare), the postoperative care instructions including pain management, activity restriction, wound care, specialist instructions and follow‐up appointments, the impact on sexual function and the potential complications associated with the surgical procedure, including infection, bleeding or other surgery‐specific risks.

Mean scores for each category increased from the pre‐survey to the post‐survey, with increases ranging from 0.9 points to 1.9 points. In the pre‐intervention scores, pre‐operative knowledge (*M* = 2.7, *SD* = 1.01) and post‐operative knowledge (*M* = 2.5, *SD* = 1.04) were ranked lowest. However, they both showed the highest increases in the post‐survey, with pre‐operative knowledge increasing to (*M* = 4.3, *SD* = 0.86), showing a mean difference of 1.6 (*t*(19) = −6.307, *p* < 0.001, 95% CI [0.979, 2.220]) and post‐operative knowledge increasing to (*M* = 4.4, *SD* = 0.68), showing a mean difference of 1.9 (*t*(19) = −7.292, *p* < 0.001, 95% CI [1.318, 2.481]). Benefits of surgery were ranked highest in the pre‐survey (*M* = 3.9, *SD* = 0.718) and saw the lowest increase in the post‐survey at 0.9 points. Inferential tests revealed statistically significant increases in each of the elements (see Table 2). Further details of the paired t‐test, Wilcoxon and sign test can be found in File S1.

**TABLE 2:** Pre‐ and post‐survey knowledge scores with statistical comparison.

Knowledge statements	Pre‐survey mean (SD)	Post‐survey mean (SD)	Mean difference	Mean SD	Standard error mean	95% CI lower	95% CI upper	*t*	*df*	*p*
The general surgical procedure	3.4 (0.68)	4.6 (0.5)	1.2	0.84404	0.18873	0.80498	1.59502	−6.902	19	< 0.001
The different surgical options and choices involved	3.3 (0.723)	4.6 (0.48)	1.3	0.86783	0.19405	0.89384	1.70616	−7.429	19	0.001
The risks of surgery	3.6 (0.88)	4.7 (0.47)	1.1	0.99765	0.22308	0.63309	1.56691	−5.082	19	< 0.001
The potential benefits of surgery	3.9 (0.718)	4.8 (0.41)	0.9	0.82682	0.18488	0.51304	1.28696	−5.107	19	< 0.001
The preoperative considerations	2.7 (1.01)	4.3 (0.86)	1.6	1.32654	0.29662	0.97916	2.22084	−6.307	19	< 0.001
The postoperative care instructions	2.5 (1.04)	4.4 (0.68)	1.9	1.24258	0.27785	1.31846	2.48154	−7.292	19	< 0.001
The impact of surgery on sexual function	3.1 (1.02)	4.3 (0.58)	1.2	1.17337	0.26237	0.65085	1.74915	−5	19	< 0.001
The potential complications associated with the surgical procedure	3.1 (1.08)	4.6 (0.587)	1.5	1.22921	0.27486	0.92471	2.07529	−5.252	19	< 0.001

*Note:* This table summarises the statistical analysis of pre‐ and post‐survey knowledge scores, highlighting mean differences, variability and significance levels across various surgical‐related knowledge statements.

### 3.3 Impact on Confidence

**FIGURE 3:**
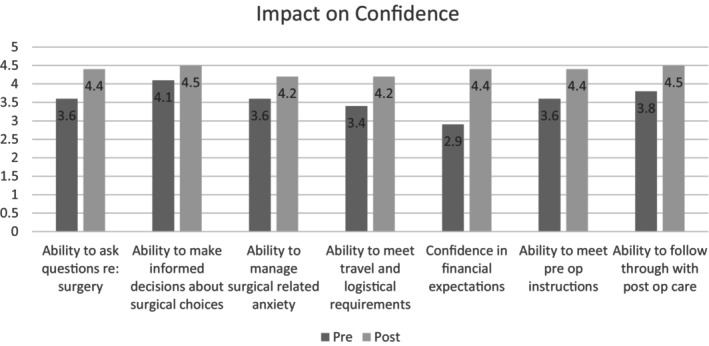
Impact on Confidence: Self‐Perceived Understanding Across Surgical Topics. This figure illustrates the statistically significant improvement in participants' self‐perceived confidence scores across diverse aspects of gender‐affirming genital surgery, using a five‐point Likert scale.

There was a statistically significant increase in self‐perceived confidence on all points assessed (Figure 3). These points were evaluated using a five‐point Likert scale that assessed participants' understanding by asking, ‘How confident are you in your ability to…’. This scale ranged from ‘Not at all’ to ‘Extremely’. The points assessed included the ability to: ask questions about the surgical procedure, make informed decisions about surgical choices, manage anxiety related to surgery, meet travel and logistical expectations related to surgery, manage financial expectations related to surgery, prepare for the surgery, including preoperative instructions, follow through with post‐operative care instructions and attend follow‐up appointments.

Mean scores for each category increased from the pre‐survey to the post‐survey, with increases ranging from 0.4 points to 1.5 points. Confidence in the ability to meet financial expectations (*M* = 2.9, *SD* = 1.05) and confidence in the ability to meet travel and logistical expectations (*M* = 3.4, *SD* = 1.05) were ranked lowest in the pre‐survey and showed the highest increases in the post‐operative survey. Confidence in the ability to meet financial expectations increased to (*M* = 4.4, *SD* = 0.75), showing a mean difference of 1.5 (*t*(19) = −5.659, *p* < 0.001, 95% CI [0.8961, 2.103]). Confidence in the ability to meet travel and logistical expectations increased to (*M* = 4.2, *SD* = 0.786), showing a mean difference of 0.8 (*t*(19) = −3.238, *p* = 0.007, 95% CI [0.1861, 1.413]). These results indicate significant improvements in participants' confidence levels following the intervention.

The ability to ask questions about the surgery and pre‐operative confidence showed high increases of 0.8 points in post‐operative results. Specifically, the ability to ask questions about surgery increased from a pre‐survey mean of 3.6 (*SD* = 0.68) to a post‐survey mean of 4.4 (*SD* = 0.68), with a mean difference of 0.8 (*t*(19) = −5.101, *p* < 0.001, 95% CI [0.349, 1.250]). Confidence in the ability to make informed decisions about surgery was ranked highest in the pre‐survey (*M* = 4.1, *SD* = 0.71) and saw the lowest increase in the post‐operative survey at 0.4 points, increasing to a post‐survey mean of 4.5 (*SD* = 0.60), with a mean difference of 0.4 (*t*(19) = −2.629, *p* = 0.039, 95% CI [−0.035, 0.835]). Inferential tests revealed statistically significant increases in each of the elements (see Table 3). Further details of the paired t‐test, Wilcoxon and sign test can be found in File S1.

**TABLE 3:** Pre‐ and post‐survey confidence scores with statistical comparison.

Confidence statement	Pre‐survey mean (SD)	Post‐survey mean (SD)	Mean difference	Mean SD	Standard error mean	95% CI lower	95% CI upper	*t*	*df*	*p*
Your ability to ask questions about the surgical procedure and postoperative care	3.6 (0.68)	4.4 (0.68)	0.8	0.96167	0.21503	0.34993	1.25007	−5.101	19	< 0.001
Your ability to make informed decisions about surgical choices	4.1 (0.71)	4.5 (0.6)	0.4	0.92957	0.20786	−0.03505	0.83505	−2.629	19	0.039
Your ability to manage your anxiety related to the surgical procedure	3.6 (0.82)	4.2 (0.52)	0.6	0.97098	0.21712	0.14557	1.05443	−3.269	19	0.008
The travel and logistical expectations related to the surgery	3.4 (1.05)	4.2 (0.786)	0.8	1.3116	0.29328	0.18615	1.41385	−3.238	19	0.007
The financial expectations related to the surgical procedure	2.9 (1.05)	4.4 (0.75)	1.5	1.29035	0.28853	0.8961	2.1039	−5.659	19	< 0.001
Your ability to prepare for the surgical procedure, including following preoperative instructions	3.6 (0.933)	4.4 (0.68)	0.8	1.15451	0.25816	0.25967	1.34033	−4.292	19	0.002
Your ability to follow through with postoperative care instructions and attend follow‐up appointments	3.8 (0.933)	4.5 (0.512)	0.7	1.06425	0.23797	0.20191	1.19809	−3.322	19	0.006

*Note:* This table summarises the statistical analysis of pre‐ and post‐survey confidence scores, providing insights into the participants' confidence levels before and after the intervention.

### 3.4 Influence on Level of Support

**FIGURE 4:**
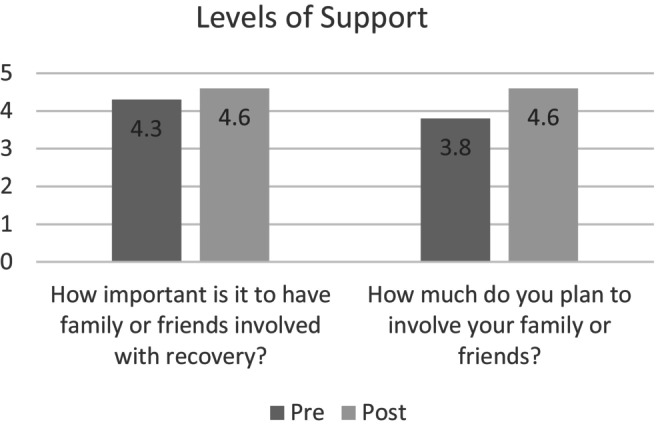
Influence on level of support: perspectives on family involvement in gender‐affirming surgery. This figure illustrates the perceived importance of involving family in gender‐affirming surgery, both pre and post the intervention, along with participants' plans to involve family or friends after the educational session.

Participants were asked four questions about their perspectives on levels of support prior to surgery (Figure 4). They were asked about their current level of family support and whether they felt comfortable discussing their surgery plans with family. Mean scores revealed that participants were moderately supported (*M* = 3.7, *SD* = 1.3), but their comfort when talking with family about surgery was lower (*M* = 3.0, *SD* = 1.5).

Both before and after the intervention, participants were asked about how important they felt it was to include family or friends in helping with recovery. Participants indicated they considered it important at baseline (*M* = 4.3, *SD* = 0.745), and this increased by 0.3 points post‐intervention to (*M* = 4.6, *SD* = 0.745), with a mean difference of 0.3 (*t*(19) = −2.349, *p* = 0.07, 95% CI [−0.1930, 0.7930]). However, this increase was not found to be statistically significant.

Participants were also asked both before and after the intervention whether they planned to involve their family or friends in their post‐surgical recovery. The mean scores increased by 0.8 points from (*M* = 3.8, *SD* = 0.961) to (*M* = 4.6, *SD* = 0.745), with a mean difference of 0.8 (*t*(19) = −4.073, *p* < 0.001, 95% CI [0.234, 1.365]). This increase was found to be statistically significant. Further details of the paired t‐test, Wilcoxon and sign test can be found in File S1 and in Table 4.

**TABLE 4:** Pre‐ and post‐survey support statements with statistical comparison.

Support statements	Pre‐survey mean (SD)	Post‐survey mean (SD)	Mean difference	Mean SD	Standard error mean	95% CI lower	95% CI upper	*t*	*df*	*p*
How supportive is your family of your decision to undergo gender affirming genital surgery?	3.7 (1.3)	NA								
Do you feel comfortable discussing your surgery plans with your family?	3.0 (1.5)	NA								
How important do you think it is to have family or friends involved in helping you recover after gender affirming genital surgery?	4.3 (0.745)	4.6 (0.745)	0.3	1.0535891	0.235589686	−0.193094879	0.793094879	−2.349	19	0.07
How much do you plan to involve your family or friends in your post‐surgical recovery?	3.8 (0.951)	4.6 (0.745)	0.8	1.20806705	0.270132005	0.234607216	1.365392784	−4.073	19	< 0.001

*Note:* This table presents the statistical analysis of pre‐ and post‐survey responses regarding support‐related statements, highlighting changes in participants' perceptions of support and involvement in their surgical recovery process.

### 3.5 Relationship With Body

**FIGURE 5:**
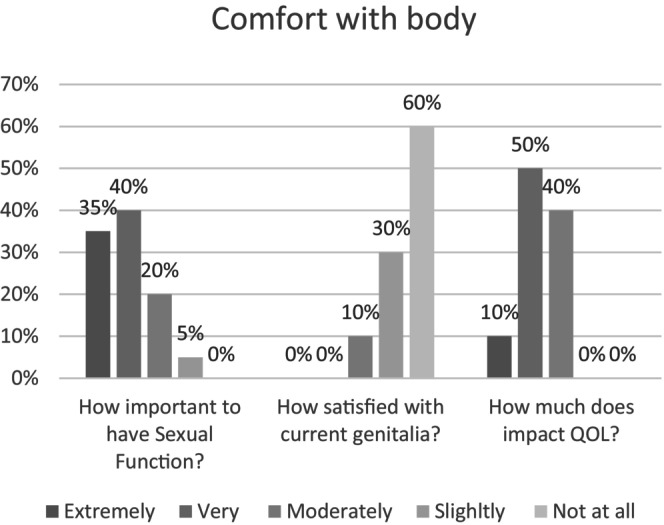
Gender dysphoria and body satisfaction. This figure provides insights into participants' experiences with gender dysphoria, the significance of maintaining sexual function and the levels of dissatisfaction with current genitals, and the impact on quality of life.

All participants consistently struggle with gender dysphoria, with 20% experiencing it all the time, 65% often, 10% half of the time and 5% sometimes. Maintaining sexual function was identified as an important outcome, with 75% of participants noting it as extremely or very important (Figure 5). Unsurprisingly, 90% of participants reported feeling extremely or very dissatisfied with their current genitals, and 60% felt it significantly or very much negatively impacted their quality of life.

### 3.6 Usefulness of Information Session

Participants were asked to rank their satisfaction with the session on a five‐point Likert scale, ranging from extremely dissatisfied to extremely satisfied. All participants reported being extremely satisfied with the information session. Additionally, 100% of participants noted that they learnt something new and would recommend the information session to a friend considering gender‐affirming surgery.

Regarding the session format, 80% of participants found the sessions in smaller groups to be ‘extremely appropriate’, with the remaining 20% feeling that it was ‘very appropriate’. Some participants provided feedback suggesting that the sessions could be split into several shorter meetings instead of one long session.

The skillset of the presenters was positively reflected in the feedback. Seventy‐five percent of participants felt the presenters were ‘extremely clear’, while the remaining 25% felt they were ‘very clear’.

Participants were asked to provide positive feedback on what they liked. Some qualitative comments included the following: one participant liked the tone and atmosphere created, stating, ‘I liked the written documents, comprehensive info, non‐judgmental environment; the tone was VERY open’. Similarly, another participant commented, ‘I liked the directness of the session and that the nurse was not afraid to discuss anatomy or potential complications. They did not seem hesitant about getting into the minutiae of surgeries’. Lastly, one participant noted how the one‐on‐one nature of the sessions meant that they could be adapted to the needs of the specific individual, saying, ‘That it was a no‐pressure, open chat and could be tailored to the individual’.

In the qualitative section, participants had no additional topics that they felt needed to be added to the content. Overall, they reported feeling that their expectations were met and generally exceeded. Interestingly, at the end of the session, 75% of participants felt the session had no impact on their desire to pursue surgery now. The remaining 25% reported that they did not have a timeline in place for pursuing surgery yet.

### 3.7 Factors Impacting Choice of Surgeon

In the post‐intervention questionnaire, participants were asked a series of open‐ended questions related to the factors influencing their choice of a surgeon for gender‐affirming surgery. The final question specifically prompted participants to identify ‘what was the most pressing factor that would influence their choice of surgeon?’. The responses were transcribed and subjected to thematic analysis.

The data were initially coded to identify recurring themes related to the decision‐making process. This process involved line‐by‐line coding, where each statement was categorised based on the underlying factors it represented. Two primary themes emerged from this analysis: *Surgeon's experience* and *Personalised approach*.

- *Surgeon's experience*: This theme, identified in 55% of participants' responses, highlighted the importance of the surgeon's track record. Key factors included the end results of surgeries, complication rates, and the number of revisions. For example, one participant noted, ‘Experience is most important to me’, reflecting a common sentiment that the surgeon's expertise is paramount.
- *Personalised approach*: Representing 15% of the cohort, this theme emphasised the importance of a surgeon's ability to offer specific techniques or approaches tailored to individual needs. Additionally, bedside manners and communication styles were highlighted by 10% of participants as critical to their decision‐making. One participant expressed, ‘Bedside manner, for me, this is reflective of how they will listen to my concerns’.

An example of this coding process is detailed in the Table 5:

**TABLE 5:** Factors influencing the choice of surgeon.

Theme	Description	Sub‐theme	Codes	Illustrative quotes
Factors impacting choice of surgeon	This theme explores the different factors that influence individuals' decisions when choosing a surgeon for gender‐affirming surgery	*Surgeon's experience*	Experience Expertise Competence Low complication rate	‘Experience, nothing wrong with new surgeons, they have to learn too but MUST be supervised by someone who has experience’ ‘Expertise, it is such a big undertaking and needs to be with a surgical team with experience and managing the procedure and complications’ ‘Experience is most important to me’ ‘The surgeon's experience, the more surgeries performed, the more experience and less complications (usually)’
		*Personalised approach*	Patient understanding Bedside manner Respect Choice in procedures	‘That I feel that they understand the needs of the patient’ ‘The techniques available matter to me as I know what I want and need’ ‘Bedside manner, for me, this is reflective of how they will listen to my concerns’ ‘Respect for transgender people’

*Note:* This table presents key themes and participant quotes on factors influencing the choice of surgeon for gender‐affirming surgery, focusing on the surgeon's experience and a personalised approach.

The analysis revealed that the most pressing factor, cited by a majority of participants (55%), was the surgeon's track record – particularly how well the end result looked and the surgeon's statistics on complications and revisions. Access to specific surgical techniques or approaches was the second most important factor for 15% of participants. Additionally, factors such as waiting times (10%), cost and insurance coverage (10%), and the surgeon's bedside manner and communication style (10%) were also significant in the decision‐making process.

## 4 Discussion

### 4.1 Understanding the Impact on Knowledge and Confidence

The increase in participants' knowledge and confidence levels post‐intervention aligns with existing literature on the positive effects of preoperative educational sessions in various healthcare domains (Falvo 2011; Casimir et al. 2014; Sandlin et al. 2013; van Driel et al. 2016). The tailored, one‐on‐one approach facilitated by nursing professionals contributed to the participants' enhanced understanding of all areas, with particular increases in knowledge about pre‐operative requirements, post‐operative recovery, changes in sexual function and complications. In addition, the research supported an increased confidence in patients ability to ask questions to surgeons, to manage their anxiety and to plan for logistical and financial expectations.

Moreover, by ensuring that patients have a better understanding of their surgical options and outcomes, their level of empowerment increases, enabling them to make informed choices, which is an important responsibility of healthcare professionals in providing high‐quality healthcare (Paterick et al. 2008; Woolf et al. 2005). The role of nurses in conveying complex medical information clearly and building trust is a key component in shared decision‐making (Chung et al. 2021) and this study played a crucial role in the success of the sessions. An opportunity for future iterations of this study would be to incorporate validated surveys or scales on anxiety and self‐efficacy and see if the education sessions have statistically significant increases pre‐ and post‐intervention.

### 4.2 Family Support and Involvement in Recovery

While participants reported moderate current family support, their comfort discussing surgery plans with family was comparatively lower. This discrepancy may indicate a need for additional interventions to facilitate open communication within familial relationships. The consistent high importance attributed to involving family or friends in recovery, coupled with a significant increase in participants planning to do so, underscores the pivotal role of support networks in the gender‐affirming journey and the benefit of education sessions in promoting this.

This study highlights the dynamics involved in balancing individual needs, familial understanding and the crucial role played by emotional support during the recovery process. One of the benefits of this study was that family members were welcome to attend the session with the patient seeking surgery. A potential avenue for further research involves evaluating the knowledge and level of support among family members both before and after the information sessions. Additionally, there is an opportunity to develop a dedicated resource for families specifically addressing gender‐affirming surgical education.

In the established research, a significant challenge that has been repeatedly identified is limited access to education for families (Kuvalanka, Weiner, and Mahan 2014; Sharek, Huntley‐Moore, and McCann 2018). Both Sharek, McCann, and Huntley‐Moore (2020) and Matsuno and Israel (2021) have undertaken the design and development of online educational resources targeting families of trans youth with the aim of increasing support and acceptance of their gender identities. However, these resources are aimed specifically at young people and provide limited information on surgical procedures or decision‐making related to surgery. Hence, an age‐appropriate session on surgical information that actively involves an in‐person component seems to be an unmet need.

### 4.3 Complexities of Gender Dysphoria and Body Image

The study sheds light on the pervasive and significant struggles with gender dysphoria among participants, emphasising the multifaceted challenges faced by gender‐diverse individuals and aligning with studies looking at the phenomenology of gender dysphoria in adults (Cooper et al. 2020). The high levels of dissatisfaction with current genitals, coupled with the impact on quality of life, underscore the complexity of body image concerns within this population.

The results indicate a need for holistic and individualised approaches to address the psychological and emotional aspects of gender dysphoria. Coyne, Yuodsnukis, and Chen (2023) discuss the importance of optimising healthcare through multidisciplinary approaches and acknowledge the intersection of mental health needs and medical and surgical needs. It stands to reason that psychological support should be embedded into the gender surgery journey before, during and after surgery.

Additionally, the emphasis on maintaining sexual function as a crucial outcome reflects the sexual priorities individuals bring to their decision‐making process, reinforcing the importance of psychosexual maturity and comprehension. It is important to note that potential negative effects on sexual function can be an outcome of these surgeries, as highlighted in both studies by Dunford, Bell, and Rashid (2021) and Frey et al. (2016).

### 4.4 Participant Satisfaction and Session Format

The 100% satisfaction rate and positive qualitative feedback on the session atmosphere, tone and the directness of discussions indicate the success of the nurse‐led, one‐on‐one format. The personalised sessions were deemed appropriate by the majority, with suggestions for shorter meetings reflecting an understanding of participants' attention spans and engagement levels.

The positive reception showcases the importance of creating a non‐judgemental and open environment where participants feel comfortable discussing sensitive topics. These findings support the assertion that individualised approaches led by nursing professionals contribute to a positive and personalised educational experience. In addition, there are notable benefits to including family members and loved ones, and this should be explored further in future research.

### 4.5 Factors Influencing Choice of Surgeon

The study's exploration of factors influencing the choice of surgeon aligns with the broader discourse on the importance of patient‐centred care in gender‐affirming surgeries. The emphasis on the track record of the surgeon, including the aesthetic outcomes and complication rates, agrees with existing literature. Ettner, Ettner, and White (2016) found that the skill of the provider was the most predominant decision‐making factor when choosing a surgeon.

The consideration of specific techniques or approaches, waiting times, cost, and communication styles indicated that there are many factors that individuals prioritise in their decision‐making process. These findings provide valuable insights for healthcare providers in tailoring their approaches to meet the nuanced preferences of gender‐diverse individuals seeking surgery.

It is important to recognise that this research was conducted in Europe which has different insurance and financial considerations than other regions. Additional research is necessary to explore the factors influencing surgeon choice across other regions.

### 4.6 Implications for Future Research and Healthcare Initiatives

This study contributes to the evolving field of gender‐affirming care by providing insights into the effectiveness of nurse‐led psychoeducation sessions. Providers of gender affirming care could replicate these sessions to better educate their patients.

The positive outcomes and participant feedback lay a foundation for future research, emphasising the need for continued exploration of individualised interventions and initiatives for family members and loved ones. Additionally, the findings underscore the importance of addressing the complex interplay between mental health, timing, psychosexual maturity and surgical decision‐making.

While this was an evaluative study that primarily drew on quantitative data in the form of pre and post intervention surveys, more research on the qualitative experiences of gender diverse patients views on seeking surgery, access barriers and unmet need would be useful.

This study is limited by a small size and could benefit from a larger sample. However, as a pilot initiative, it shows positive potential for more generalisable results in a larger sample.

## 5 Conclusion

In conclusion, this study advances our understanding of the impact of tailored psychoeducation sessions on knowledge, confidence and support among gender‐diverse individuals considering gender‐affirming genital surgery. The findings emphasise the need for holistic and individualised approaches in gender‐affirming care, providing valuable implications for both research and healthcare practices in the area of healthcare. This study suggests that nurse‐led initiatives are a sustainable and effective solution to improving knowledge and confidence for those seeking surgery and should be considered by gender services globally.

## Author Contributions

S.K., P.F., D.O.S., and K.N. made substantial contributions to conception and design, or acquisition of data or analysis and interpretation of data; S.K., P.F., D.O.S., and K.N. Involved in drafting the manuscript or revising it critically for important intellectual content; S.K., P.F., D.O.S., and K.N. Given final approval of the version to be published. Each author should have participated sufficiently in the work to take public responsibility for appropriate portions of the content; S.K., P.F., D.O.S., and K.N. Agreed to be accountable for all aspects of the work in ensuring that questions related to the accuracy or integrity of any part of the work are appropriately investigated and resolved.

## Conflicts of Interest

The authors declare no conflicts of interest.

## Peer Review

The peer review history for this article is available at https://www.webofscience.com/api/gateway/wos/peer‐review/10.1111/jan.16532.

## Statistical Statement

The author(s) affirm that the methods used in the data analyses are suitably applied to their data within their study design and context, and the statistical findings have been implemented and interpreted correctly.

## Supporting information


File S1.

## Data Availability

The data that support the findings of this study are available from the corresponding author upon reasonable request.
