# Supplementary figures and images for: An Evaluative Study of a Nurse‐Led Surgical Information Initiative for Gender Diverse Individuals Seeking Genital Surgery
Source: J Adv Nurs. 2024 Oct 19;81(7):3834–47. doi: 10.1111/jan.16532 (PMC12159365; doi:10.1111/jan.16532)

## Supplementary File 1:

##
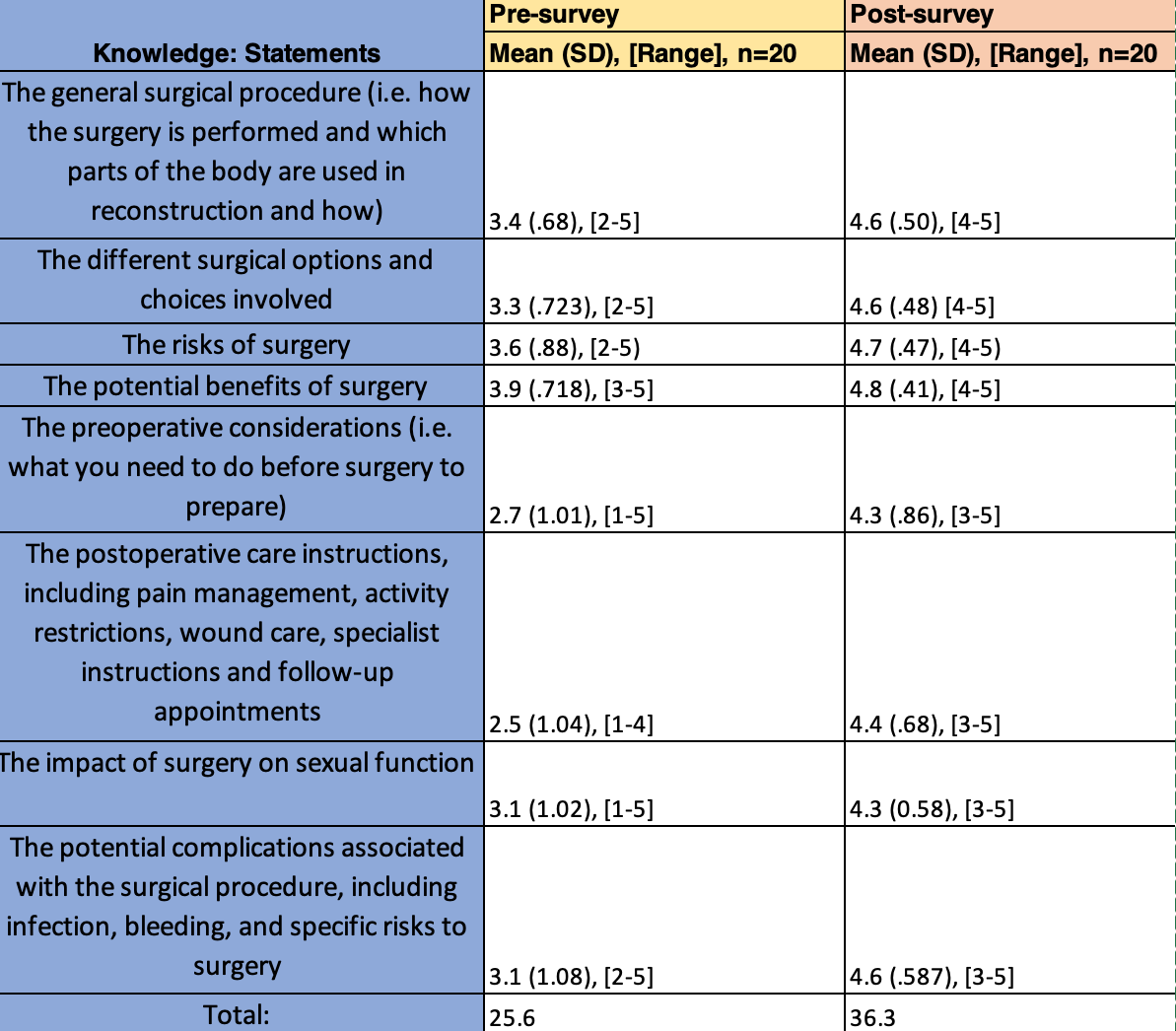
Knowledge Scores


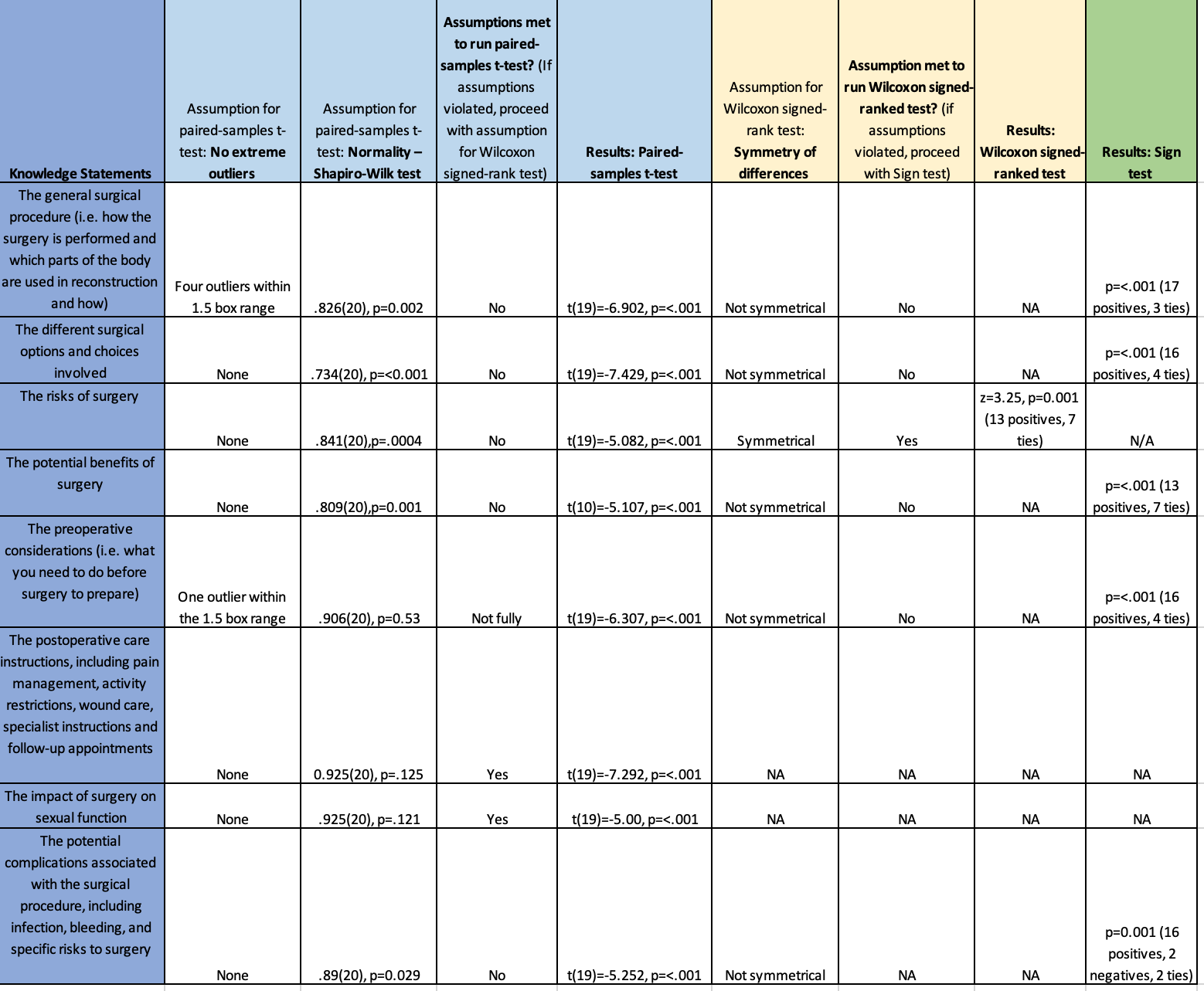


##
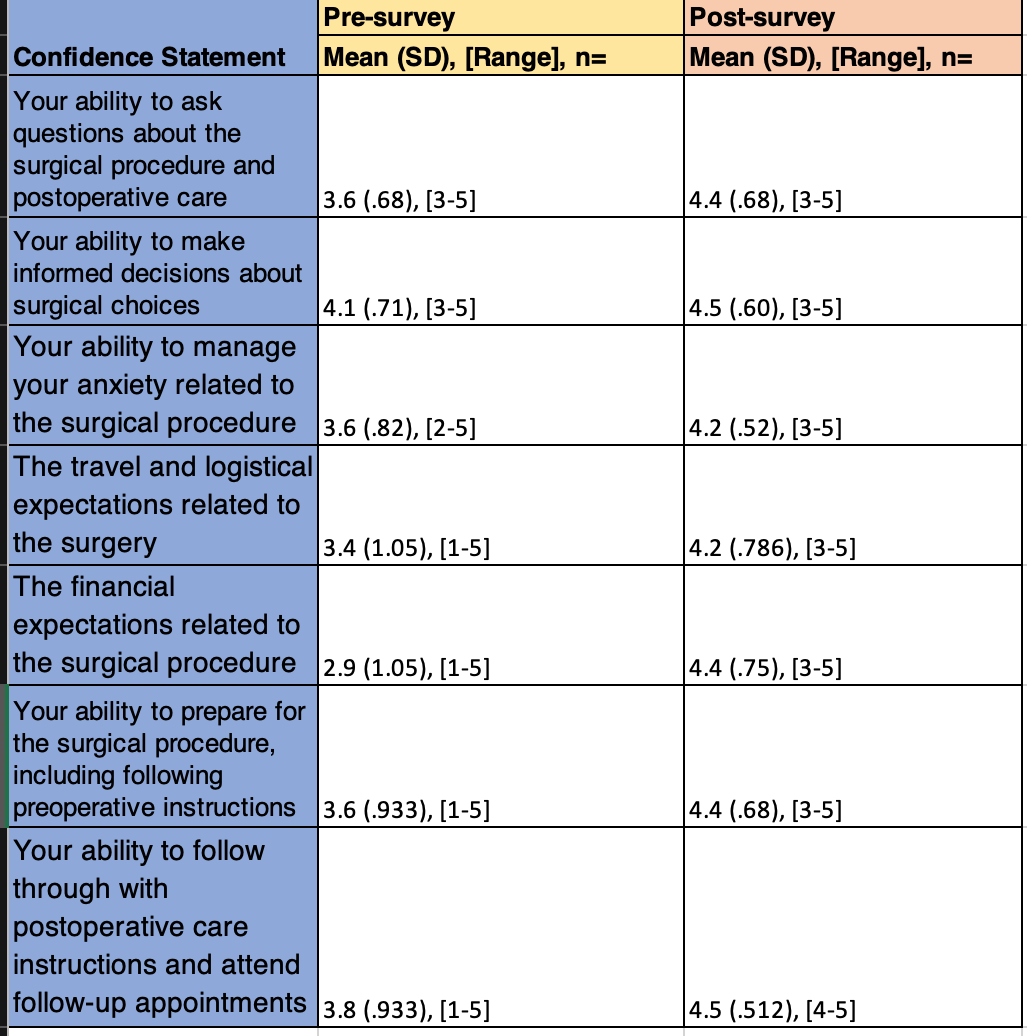
Confidence Scores


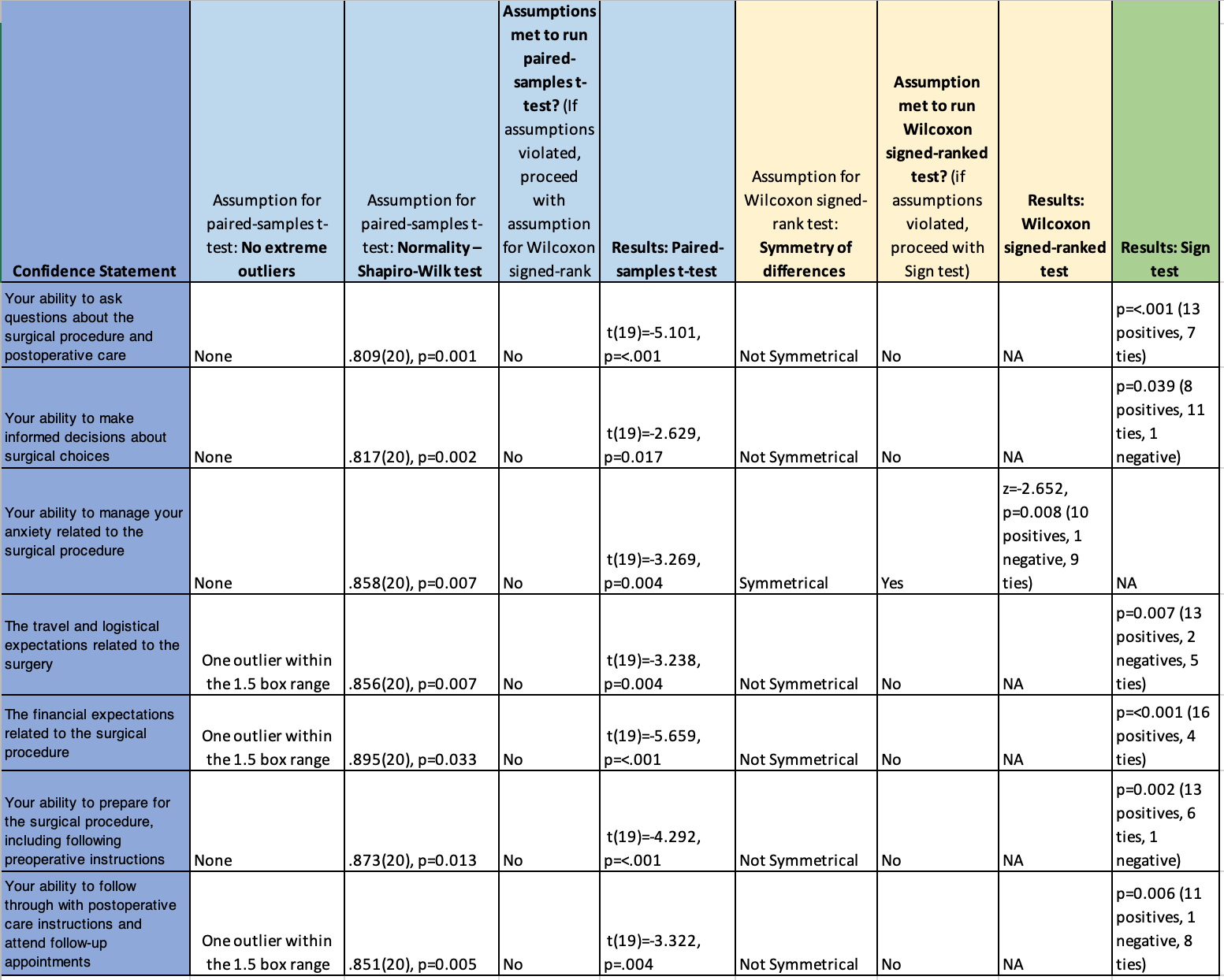


##
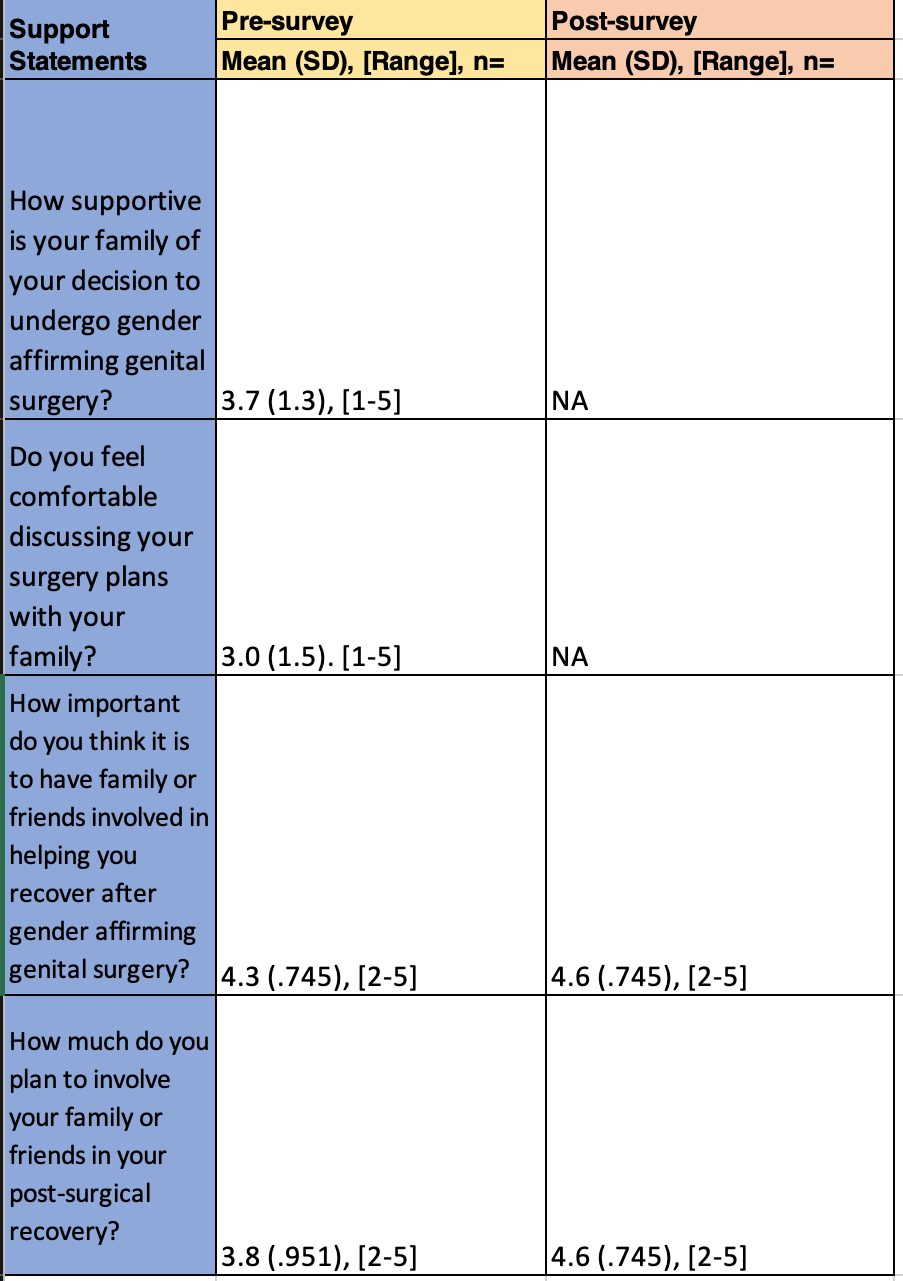
Support Scores


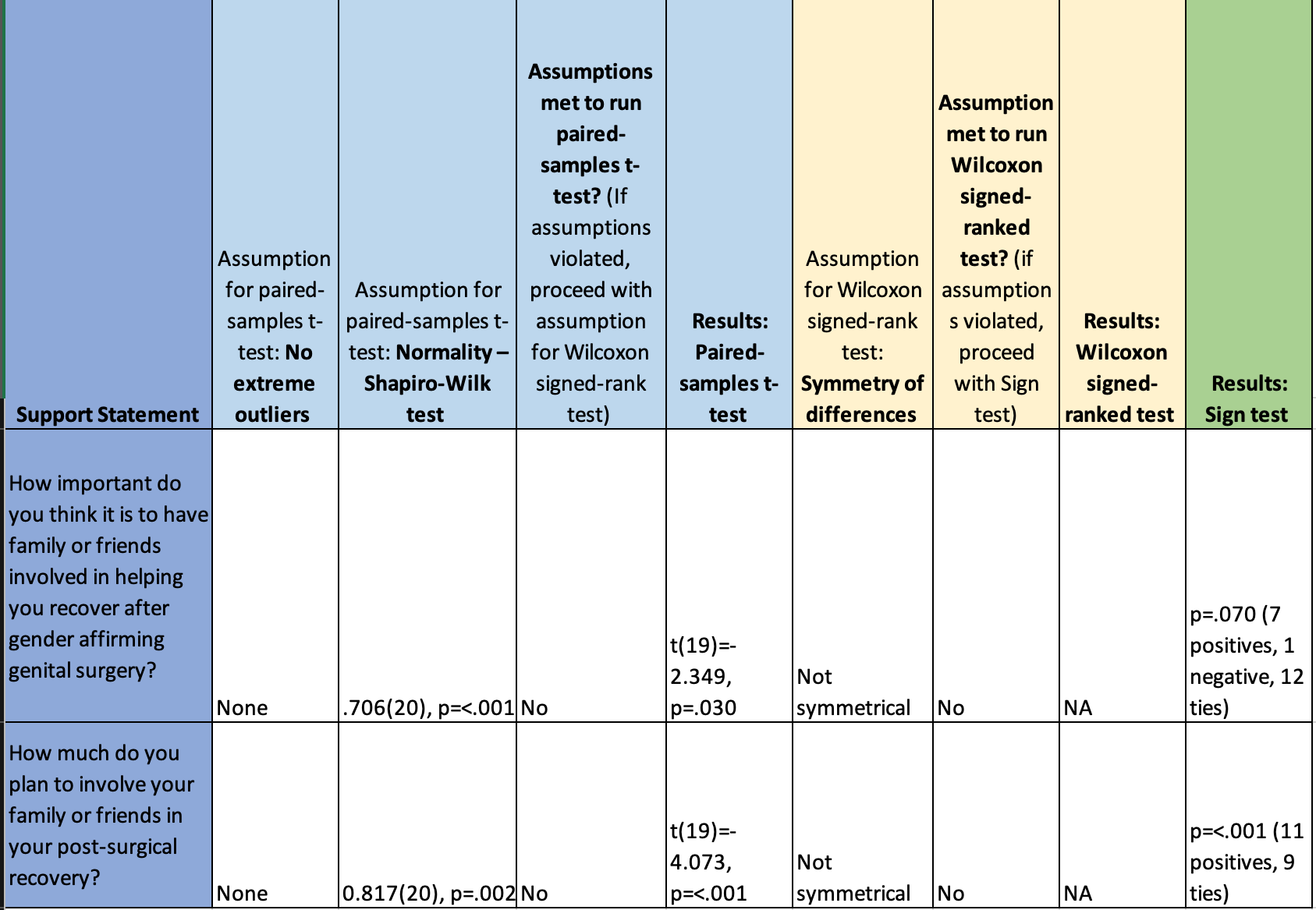

Supplement: Supplementary file 1 — File S1. [file JAN-81-3834-s001.docx]
